# Conventional Cognitive Behavioral Therapy Facilitated by an Internet-Based Support System: Feasibility Study at a Psychiatric Outpatient Clinic

**DOI:** 10.2196/resprot.6035

**Published:** 2017-08-24

**Authors:** Kristoffer NT Månsson, Hugo Klintmalm, Ragnar Nordqvist, Gerhard Andersson

**Affiliations:** ^1^ Department of Psychology Stockholm University Stockholm Sweden; ^2^ Department of Adult Psychiatry PRIMA Barn- och Vuxenpsykiatri Stockholm Sweden; ^3^ Centre for Psychiatry Research Department of Clinical Neuroscience Karolinska Institutet Stockholm Sweden; ^4^ Division of Psychology Department of Clinical Neuroscience Karolinska Institutet Stockholm Sweden; ^5^ Department of Behavioural Sciences and Learning Linköping University Linköping Sweden

**Keywords:** cognitive behavioral therapy, Internet-treatment, psychiatry, blended therapy

## Abstract

**Background:**

Cognitive behavioral therapies have been shown to be effective for a variety of psychiatric and somatic disorders, but some obstacles can be noted in regular psychiatric care; for example, low adherence to treatment protocols may undermine effects. Treatments delivered via the Internet have shown promising results, and it is an open question if the blend of Internet-delivered and conventional face-to-face cognitive behavioral therapies may help to overcome some of the barriers of evidence-based treatments in psychiatric care.

**Objective:**

We evaluated the feasibility of an Internet-based support system at an outpatient psychiatric clinic in Sweden. For instance, the support system made it possible to send messages and share information between the therapist and the patient before and after therapy sessions at the clinic.

**Methods:**

Nine clinical psychologists participated and 33 patients were enrolled in the current study. We evaluated the usability and technology acceptance after 12 weeks of access. Moreover, clinical data on common psychiatric symptoms were assessed before and after the presentation of the support system.

**Results:**

In line with our previous study in a university setting, the Internet-based support system has the potential to be feasible also when delivered in a regular psychiatric setting. Notably, some components in the system were less frequently used. We also found that patients improved on common outcome measures for depressive and anxious symptoms (effect sizes, as determined by Cohen *d*, ranged from 0.20-0.69).

**Conclusions:**

This study adds to the literature suggesting that modern information technology could be aligned with conventional face-to-face services.

## Introduction

During the last decade, there has been a growing interest in alternative ways of delivering psychological treatments. The development of Internet-delivered interventions targeting common psychiatric and somatic disorders is one promising method [1,2]. Therapist-guided Internet-delivered treatments based on cognitive behavioral therapy (ICBT) have commonly shown promising effects in studies of both research studies (efficacy) [3], and in more clinically representative settings (effectiveness) [4]. A growing body of evidence suggests similar outcomes of ICBT and conventional face-to-face cognitive behavioral therapies (CBT) [2], with therapist-guided ICBT being less time-consuming for the clinician. Using the Internet to deliver health care may open new avenues to treatment, especially in societies where the distance to care is far away. Thus, ICBT has the potential to increase access to evidence-based psychological treatment [3].

In primary or psychiatric care, there may be some obstacles of providing conventional CBT delivered face-to-face. For instance, therapists may be prone to drift away from implementing effective interventions (ie, therapist drift) [5], and they may also fail to adhere to evidence-based treatment manuals [6]. One way to overcome such obstacles could be to provide computer-assisted support in therapeutic work [7]. In a previous study, we developed an Internet-based support system to facilitate the delivery of conventional CBT [8]. The basic idea of the system is to support the delivery of CBT in a clinical setting where the therapist meets their patients face to face. By providing support, our objective was to improve the delivery of regular treatment components present in CBT, for example, homework assignments. A potential strength of the approach is that it conceptually shifts the focus of research away from specific digital interventions towards the system level (ie, capable of delivering many interventions). The approach also highlights the potential impact of introducing digital communication channels in face-to-face psychotherapy. The initial study showed some promising findings in terms of user experiences (eg, the ease of providing written information as a complement to the therapy sessions), and we observed reliable reductions of depressive and anxious symptoms. The study was conducted in a university setting, and there is a need to test the support system in clinical psychiatric care (eg, with a more severe clinical population and across different disorders).

This feasibility study aimed at evaluating the experiences and effects of an Internet-based support system used as an adjunct to conventional CBT delivered face to face. The system was designed to support the delivery of face-to-face CBT and not replace in-session treatment activities. The system was used for communication between therapy sessions, sharing media, and clarifying homework assignments [8]. Clinicians and patients were recruited from a psychiatric clinic in Sweden, and the users were given access to the support system during 12 weeks. At follow-up, we evaluated support system usability and technology acceptance. Moreover, self-report questionnaires targeting clinical symptoms at baseline and 12-week follow-up were also administered.

## Methods

### Procedure

Nine clinical psychologists participated as therapists in the study. The clinicians were asked to recruit patients from the clinic in accordance with the standard procedures at the clinic. In line with the ethics committee agreement (ID: 2013/452-31), all patients were informed about the objectives of the study via a document printed on paper and asked to provide written informed consent before inclusion. All patients answered questionnaires regarding clinical and demographic characteristics via the Internet. After inclusion, the clinician registered the patient in the support system and distributed an online follow-up survey after 12 weeks access of the support system. Mean time between assessments was 91 days (range 61-116).

### The Support System

The Internet-based support system used in this study was previously developed and tested in a pilot study conducted in a university clinic setting [8]. Also, the support system has been used in audiologic practice in supporting first-time hearing aid clients [9]. In brief, the support system was accessible via personal computers through an encrypted secured socket layer connection to the Internet. Users were assigned personal login identifications via email. Also, to increase security, an additional temporary password was sent via mobile phone text messages at each attempt to log on.

The support system facilitated a variety of functions and the therapists decided themselves on how to use the content, tailored to the patients’ needs, and components included communication between sessions with the ability to send mobile phone text messages. Via the support system, the therapist also had the opportunity to send mobile phone text messages to the patients. The support system included a library that mainly provided text documents, but also other media such as audio and movies were made accessible. These resources were compiled primarily from prior studies on Internet-delivered CBT for anxiety and depression [10], and they were not presented as separate treatments but rather as part of the face-to-face treatment (eg, as online handouts). Topics covered in the online handouts contained supplemental information on CBT, such as behavioral activation, activity scheduling, exposure therapy, common cognitive biases, and maintenance of avoidance via safety behaviors. We also provided some audio files, such as relaxation instructions. In addition, the support system included common questionnaires and forms used in homework assignments, such as guides to create a fear hierarchy, daily thought records, and sleep diaries. For an overview of all the functions, see Table 1.

**Table 1:** List of included components in the Internet-based support system.

	Editable by^a^	
	Patient	Clinician
Formulate homework assignments	No	Yes
Library (sharing content)	No	Yes
Registration forms	Yes	Yes
Sending internal messages	Yes	Yes
Sending mobile phone text messages	No	Yes
Setting an agenda	No	Yes
Setting goals	Yes	Yes
Uploading files to the patient’s personal library	Yes	Yes
Uploading new files to the library	No	Yes
Writing memos	Yes	Yes

^a^The term editable means that the user was able to edit, add, or delete content within that specific component/function in the support system.

#### Technical Issues

During the study period, we had one main technical problem with the support system. As a way of warranting the security of the support system, it was designed to automatically log out inactive users (as determined by no clicks with the pointer). First, the support system automatically disconnected users after 10 minutes of inactivity. A number of users gave us feedback that text had been lost due to this function (eg, while writing a long message exceeding 10 minutes, the user was incorrectly disconnected). Consequently, we increased this time frame to 40 minutes during the study period.

Before study initiation, we invited a group of clinicians to a 2-hour workshop offering a brief overview of the support system. Also, the clinicians logged in to the system and were instructed to complete five tasks in order to acquire some knowledge on basic functions in the support system, for example, log in to the system, create a new user (patient), send the patient a message, share a file from the library with the patient, as well as a registration form for behavioral experiments.

### Participants and Recruitment

The included clinicians’ professional status and demographic characteristics are presented in Table 2. The clinicians volunteered and did not receive any compensation for their participation.

During the study period, 52 patients were registered in the support system. However, data from 4 patients were missing at the baseline assessment, 12 patients were missing at follow-up, and for 7 patients assessment data were completely missing (ie, both at baseline and follow-up). In total, 29 patients contributed with complete data from the pre- and posttreatment assessments. The patients’ demographic characteristics and computer experience at baseline are presented in Table 3. Participants self-rated their level of experience of using computers on a 5-point Likert scale, ranging from 0 (very limited) to 4 (very much). We did not include any clinical interview in order to determine diagnostic criterion and comorbidity. The patients received treatment but no compensation for participating in the study.

**Table 2:** Demographic and professional characteristics of the clinicians (n=9).

Characteristics		
Age in years, mean (median, SD, range)		37.78 (35, 9.2, 28-54)
Year as licensed psychologist, mean (median, SD, range)		2.44 (2, 2.5, 0-7)
Sex, female, n (%)		3 (33.3)
**Professional status, n (%)**		
	Pre-licensed under supervision	4 (44.4)
	Licensed clinical psychologist	5 (55,6)
**Clinical work (% per month), n (%)**		
	0-25%	3 (33.3)
	26-50%	1 (11.1)
	76-100%	5 (55.6)

**Table 3:** Demographic and clinical characteristics of the patients (n=45).

Characteristics		
Age in years, mean (median, SD, range)		30.58 (28,10.6, 18-60)
Work time (% per month), mean (median, SD, range)		36.11 (40, 30.7, 0-100)
Sex, female, n (%)		36 (80.0)
Having children, n (%)		29 (64.4)
**Computer experience, n (%)**		
	Less	11 (24.4)
	More	34 (75.6)
**Educational status, n (%)**		
	<High school	22 (48.9)
	>High school	23 (51.1)

The patients were either recruited from an existing wait-list at the clinic or were currently undergoing a conventional CBT at the clinic. In order to receive treatment at the psychiatric clinic, the patients had to be over 18 years of age. Eligible patients in this study were required to have some computer experience (ie, being able to handle their bank account via the Internet) and have access to a computer and mobile phone during the study period. Patients not considered eligible, or denied participation in the study, were offered conventional face-to-face CBT in line with routines at the clinic.

All procedures contributing to this work comply with the standards of the national ethical committee and with the Helsinki Declaration of 2008.

### Cognitive Behavioral Therapy

This study did not follow a manualized CBT protocol, nor did all the clinicians receive clinical supervision as part of the study. The clinicians tailored the CBT according to their patient’s needs (eg, based on cognitive case formulation or behavior analysis) and each clinician-patient pair individually decided how to use the Internet-based support system during the treatment.

We evaluated the use of the support system during a period of 12 weeks. Therefore, our assessments at baseline and 12-week follow-up were not fixed at pre- and posttreatment (ie, at baseline, some patients had already started CBT, and for some patients the CBT was not terminated at the 12-week follow-up).

### Support System Usability

For all the users (ie, clinicians and patients), we monitored the number of logins, the total time spent logged in, as well as the number of messages sent within the support system. After 12 weeks of accessing the support system, we evaluated the users’ experiences. We also asked questions targeting specific functions within the support system, for example, how often the participant read and downloaded text documents, listened to audio files from the library, set goals for the treatment, asked questions, and requested guidance via internal messages. The questions were rated on a 6-point scale ranging from never to very often. In addition, the clinicians were also asked to rate for how many of their patients the features in the support system had been, or would have been, relevant for their patients in their regular clinical practice, ranging from no one, less than 50%, more than 50%, or for most patients.

### Technology Acceptance, Perceived Usefulness, and Ease of Use

We used 19 questions targeting usability of the Internet-based support system. The questions were adopted from questionnaires of technology acceptance [11], perceived usefulness, and perceived ease of use [12] and were translated into Swedish. We used only a sample of questions and customized them to fit the current study. All questions were rated on a 7-point Likert scale ranging from “Strongly disagree” to “Strongly agree.” All participants were asked to answer these questions (ie, both clinicians and patients).

### Clinical Outcome and Quality of Life

The Beck Anxiety Inventory (BAI) [13] and the Generalized Anxiety Disorder Screener-7 (GAD-7) [14] were used both at baseline and as outcome measures of anxiety symptoms. Both questionnaires have been shown to have excellent internal consistency (Cronbach alpha >.90) [13,14]. The Montgomery Åsberg Depression Rating Scale‒self-rating version (MADRS-S) [15] and the Patient Health Questionnaire-9 (PHQ-9) [16] were used to measure symptoms of depression and suicidality. MADRS-S and PHQ-9 also have excellent internal consistency (alpha >.89) [15,16]. In the MADRS-S, suicidality was defined as a score of at least three points on item 9. Similarly, patients scoring one point (or above) on item 9 on the PHQ-9 were also considered suicidal in this study.

In addition to change in symptoms of anxiety and depression, the Quality of Life Inventory (QOLI) [17] was administered both at baseline and at 12-week follow-up. QOLI has shown good to excellent internal consistency (alpha >.77) in a clinical population with both anxious and depressive disorders [18]. In agreement with our previous studies [3], all self-report questionnaires were administered via a secured Internet-based platform.

### Data Analysis

The STATA v13.1 statistical software for Mac OS X (StataCorp) was used to analyze the data. We evaluated user experiences across patients with high versus low activity in the support system and dichotomized high versus low frequent users by performing a median split on number of times the patients accessed the support system (ie, ≥12 defined high users). Differences between users (ie, low versus high activity) groups (ie, clinicians versus patients) were analyzed using logistic regression.

We also performed analyses on clinical outcome of anxious and depressive symptoms. Similarly, quality of life was measured at baseline and 12-week follow-up. In order to account for dependency in the data (ie, longitudinal clinical outcomes), we used generalized estimating equations (GEE) with an exchangeable correlation structure, assuming that all missing data were completely at random [19]. Outcomes are presented as coefficients or odds ratios (OR). Within-group effect sizes were calculated based on the pooled standard deviation and correlation between time points, expressed as Cohen *d* with 95% confidence intervals. Furthermore, we also investigated if the number of times accessing the support system was associated with change in the patient’s symptoms of anxiety and depression.

As a way to control for multiple comparisons, we performed Bonferroni corrections within each sector of the analyses (ie, one sector corresponds to support system usability, and another was clinical outcome).

Furthermore, we explored what time of the day the patients accessed the support system. Specifically, we were interested in the proportion of logins made after the clinic was closed (ie, before 8 a.m. and after 5 p.m.).

## Results

### Support System Usability

#### Clinicians

The mean number of times the clinicians accessed the support system during the 12-week period was 94 (SD 54, median 89), and across all the clinicians the average time logged in to the support system was 1008 minutes (16.8 h, SD 784 min, median 770 min). On average, 64 messages were sent per clinician (SD 25, median 62, range 17-100). Moreover, the mean number of sent mobile phone text messages was 32 (SD 14, median 35, range 9-51).

As shown in Table 4, the clinicians’ ratings of usability demonstrate how often specific components were assigned to the patient, as well as the proportion of patients for whom this component was considered relevant in the therapeutic work. For example, sharing forms and studying information in the library for own professional development were on average used 2.8 times (ie, less used than “sometimes”). Yet, most of the clinicians rated these functions to be relevant for more than 50% of their patients.

**Table 4:** Clinicians’ (n=9) evaluation of support system usability on a 6-point Likert scale (0=never and 5=very often), sorted by mean values.

Questions			The component was relevant for n patients, n (%)	
	Mean	SD	Less than 50%	More than 50%
Sending reminders via mobile phone text messages	4.11	1.0	3 (33.3)	6 (66.7)
Shared documents, images, and audio files via the library	4.00	1.0	3 (33.3)	6 (66.7)
Answered questions	3.89	1.0	4 (44.4)	5 (55.6)
Providing support and encouragement	3.89	0.9	4 (44.4)	5 (55.6)
Formulated homework assignments	3.78	1.1	5 (55.6)	4 (44.4)
Provided psychoeducation from the library	3.44	1.9	4 (44.4)	5 (55.6)
Reading the patients reports on homework assignments	3.44	0.7	5 (55.6)	4 (44.4)
Asked for feedback on information in the library	3.22	1.2	7 (78.0)	2 (22.2)
Examined the patient work with homework	3.11	0.9	5 (55.6)	4 (44.4)
Studied information from the library for own professional development	2.88	1.3	4 (44.4)	5 (55.6)
Distributed registration forms	2.78	1.5	4 (44.4)	5 (55.6)
Reviewed homework assignments reported by the patient	2.78	1.3	7 (78.0)	2 (22.2)
Worked with assignments from the library	2.44	1.2	8 (89.0)	1 (11.1)
Corrected and revised homework assignments	2.44	1.4	6 (66.7)	3 (33.3)
Formulated goals for therapy	1.88	1.2	7 (78.0)	2 (22.2)
Setting an agenda	1.44	1.3	7 (78.0)	2 (22.2)
Play audio during the therapy session	0.11	0.3	9 (100)	0 (0)

**Table 5:** The patients’ (n=33) evaluation of support system usability on a 6-point Likert scale (0=never and 5=very often), sorted by mean values.

Questions	Mean	SD	Median
Proportion of completed homework assignments	3.27	1.3	4
Accessed your psychologists formulations of homework assignments	3.18	1.6	4
Provided information about the progress of your homework assignments^a^	2.33	1.7	3
Asked for guidance via internal messages^a^	2.18	1.8	3
Answered forms	2.15	1.7	3
Downloaded and saved information on your computer	1.91	1.7	2
Reading information from the library	1.88	1.6	2
Reading and reviewed treatment goals during the therapy	1.76	1.7	1
Printed documents	1.42	1.7	1
Reading the agenda	1.21	1.6	0
Saved your own therapy-related information (text and/or images) in your personal library	1.03	1.4	0
Listened to audio files	0.90	1.4	0
Wrote notes regarding questions to discuss with your psychologist	0.33	0.8	0
Wrote memos	0.30	0.8	0

^a^Indicating differences between the high versus low frequent users.

#### Patients

Across 12 weeks of access, the patients’ average number of logins was 14 (SD 15.3, median 11, range 1-95), and they (n=49) spent on average 92 minutes (SD 157, median 42) on the support system. One patient was an outlier and spent more than 1000 minutes logged into the support system. After excluding this outlier, the average number of minutes was reduced to 72 (SD 72, median 40), which corresponds to an average of 6 minutes of access per week (72/12) and patient. In addition, the patients sent on average 6 messages to their therapist (SD 10, median 3, range 0-58), although there is a large variation across users.

The patient’s usability ratings of specific components in the support system are presented in Table 5. High and low frequent users ratings differed significantly on two items: (1) providing information about the progress homework assignments (high users mean 3.1, SD 1; low users mean 1.6, SD 2; β=0.64, *Z*=2.40, *P*=.02), and (2) asked for guidance via internal messages (high users mean 3.1, SD 1; low users mean 1.2, SD 2; β=0.70, *Z*=2.71, *P*=.002). However, after controlling for multiple comparisons (ie, Bonferroni correction) the differences were not significant.

### Technology Acceptance, Perceived Usefulness, and Ease of Use

The clinician and the patient ratings of technology acceptance, perceived usefulness, and ease of use are shown in Table 6. The clinicians and the patients rated two items significantly differently. First, the clinicians were more motivated to use the support system after the study termination (β=0.68, *Z*=2.10, *P*=.036). Second, the patients, relative to the clinicians, highlighted that the support system reminded them about tasks to complete in the support system (β=–0.50, *Z*=2.37, *P*=.018). However, by controlling for multiple comparisons, these differences were not significant.

### Clinical Outcome and Quality of Life

Total scores on the BAI, MADRS-S, and PHQ-9 decreased from baseline to 12-week follow-up, yet the GAD-7 only showed a trend towards statistical significance. Moreover, quality of life, as measured by QOLI, increased over time (see Table 7).

Suicidal ideations, as measured by MADRS-S item 9, decreased by 14% from baseline to follow-up (OR 0.86, *Z=* 2.05, *P=*.040). However, the scored item on suicidal ideation in PHQ-9 did not change over time (OR 0.89, *Z*=1.43, *P=*.152). With the exception of change on MADRS-S suicidality and QOLI, the other results on clinical symptoms remained statistically significant following Bonferroni correction (*P<*.05).

We did not find that the number of times accessing the support system was associated with any change in clinical symptoms of anxiety or depression. We found that 30.52% (420/1376) of the patients’ logins were made after working hours at the clinic.

**Table 6:** Questionnaire targeting technology acceptance and ease of use of the support system. Ratings provided on a 7-point Likert scale (1=strongly disagree to 7=strongly agree).

Questions	Patients (n=33)		Clinicians (n=9)	
	Mean	SD	Mean	SD
1. Using the platform improves the quality of the work I do	4.09	1.9	5.22	1.0
2. The platform enables me to accomplish tasks more quickly	3.61	1.9	3.56	1.6
3. The platform increases my productivity in my work with the therapy	3.91	1.9	4.00	1.4
4. The platform improves my work and the things I need to do in therapy, such as homework assignments and practical exercises	4.58	2.0	4.56	1.0
5. Using the platform can increase my effectiveness working with the therapy	4.09	1.8	4.44	0.9
6. Using the platform makes it easier to do my tasks	4.70	2.0	4.11	1.6
7. Learning to operate the platform is easy for me	5.67	1.9	5.00	1.4
8. Performing an operation in the platform always leads to a predicted result	4.12	1.8	3.22	1.5
9. The platform has a clear interface that helps me do what I want	4.52	1.9	3.22	1.1
10. The platform is flexible and easy to interact with	4.33	1.8	3.11	1.8
11. It is easy for me to become skillful at using the platform	4.42	1.9	4.89	1.0
12. Overall, I find the platform easy to use	5.30	1.9	4.67	1.6
13. I feel confident in finding information in the platform	5.12	1.8	4.22	1.3
14. I feel confident in receiving and sending messages	5.24	1.8	5.33	1.6
15. I feel confident in downloading files	5.12	2.1	4.78	1.8
16. The platform was visually appealing	3.58	1.9	3.78	1.8
17. The platform reminds me about tasks to complete	4.79	2.1	2.67	1.9
18. The organization of information in the platform is clear	4.45	1.9	3.44	2.0
19. I would like to use the platform on a regular basis in the future	4.36	2.3	6.44	0.9

**Table 7:** Generalized estimating equations (GEE) regarding clinical symptoms and quality of life at baseline, and 12-week follow-up (total N=33 patients in the GEE; 29 contributed with complete data from baseline to follow-up).

Measure	Pre, mean (SE)	Post, mean (SE)	Coefficient	*Z*	*P*	Cohen *d*^a^ (95% CI)
BAI	20.54 (1.7)	15.82 (1.8)	-4.72	-2.88	.004	0.45 (0.1 to 0.8)
GAD-7	10.35 (0.8)	9.15 (0.9)	-1.20	-1.77	.077	0.20 (-0.1 to 0.5)
MADRS-S	20.96 (1.3)	18.18 (1.4)	-2.78	-2.61	.009	0.32 (0.0 to 0.6)
PHQ-9	13.32 (0.9)	9.19 (1.0)	-4.13	-3.68	<.001	0.69 (0.2 to 1.1)
QOLI	-0.37 (0.3)	0.03 (0.3)	0.40	2.12	.034	-0.18 (-0.4 to 0.1)

^a^Effect sizes (Cohen *d*) were calculated on observed data.

## Discussion

### Principal Findings

The aim of this feasibility study was to test an Internet-based support system in a clinical psychiatric setting with a focus on both clinician and patient experiences, and also including patient outcomes. Overall, we found that clinicians, as compared to the patients, rated some functions of the support system as more useful and that ratings by patients tended to be fairly low for some functions. We also asked clinicians to rate the proportion of patients for which the components of the support system would be useful. Less than half of the clinicians rated that the components would be useful for more than half of their patients. As there were few clinicians in the study, these estimates should be interpreted with caution but at least they signal that some functions, like sending reminders and sharing documents, may be appealing to clinicians in their work. At the same time, using the support system to formulate therapy goals, agenda setting, and playing audio files were barely used by the clinicians. Overall, usefulness ratings, ease of use, and technology acceptance varied but were fairly high for some items. Moreover, 30% of the times the patients accessed the support system were after working hours at the clinic. This indicated that this support system also has the potential to increase the availability of psychiatric care. In line with a large body of literature on the effects of CBT and ICBT, symptom ratings decreased over the study period.

This feasibility study raises many questions. First of all, the support system tested in our first study [8] appears to work when delivered in a more regular psychiatric setting with regular clinicians and patients [20]. Yet, it is important to keep in mind that a few specific functions within the system were rarely used by the clinicians. We hesitate to refer to this study as an effectiveness study as use of the support system per se was not part of regular practice, and we introduced and tested the system simultaneously. In the first study [8], we had a smaller sample and used interviews to gather information on experiences of clinicians and patients. In this study, we investigated differences between high versus low activity users and differences between the clinicians and the patients. No difference turned out to be statistically significant after controlling for multiple comparisons but indicated that the high frequent users more often sent messages to their therapist.

A second aspect to discuss relates to attitudes towards technology use and preferences (eg, [21,22]). There is a growing literature on these topics relating to ICBT, but far less work on the use of technology within face-to-face CBT, sometimes referred to as blended treatments [23], has been conducted. In addition, a recent stakeholder survey indicated that blended treatments are rated as more acceptable than ICBT with less therapist contact [24]. We expect more studies to appear in the field of blended interventions [23]. One recent example was a study on depression in which a mobile phone app was used [25]. Moreover, in this study we did not really focus on the technical aspects of the system, and there has been increased interest in the use of novel technologies and how they can be best incorporated and correctly described in digital health interventions [26].

Third, what can we expect to achieve with the support system? There is emerging literature on knowledge acquisition in CBT [27], and we believe that the support system can serve as a facilitator for patients when they learn more about themselves and the treatment presented. This might not necessarily lead to better outcomes in the short run but is also unlikely to lead to worse outcomes. In the long run, it is possible that the enhanced learning and support provided by the system could help to prevent relapse.

Fourth, this study raises questions regarding training of therapists and adherence to treatment manuals. It is possible that clinicians with less training can benefit more from blending information technology with face-to-face services. There are examples of using computerized support [7] with good outcomes, but to the best of our knowledge, there is a lack of controlled trials testing if clinicians with less training can perform as well as more skilled clinicians if they work with a support system. More experienced and well-trained clinicians may also be more effective if tasks can be delegated to the computer (eg, handling outcome measures).

### Limitations

It is important to keep in mind that this study is limited by a number of factors. First, the within-group design limits any causal inferences, and we cannot answer whether or not the support system made any substantial, positive or negative, contributions beyond the effect of conventional CBT. However, in this feasibility study (without a predefined feasibility criteria) we focused on usability and technology acceptance at an outpatient psychiatric clinic. Second, the clinicians decided whether or not to ask a specific patient about participating in the study (ie, possible self-selection bias). Consequently, it is possible that the outcome of the study is affected by confounding by indication. Third, in terms of CBT interventions, we cannot demonstrate the specific interventions the clinicians delivered. Moreover, we did not measure the therapist’s competence in delivering CBT. Nevertheless, by the use of the current support system we were able to monitor the use of some fundamental CBT components (eg, that the clinicians provided homework assignments). Fourth, the number of patients lost to follow-up may be an important sign of dissatisfaction. Nevertheless, it is plausible that this was related to issues regarding procedure of the study (eg, the clinicians were primarily responsible for initiating the follow-up assessments). In our previous study in a university setting, we had no missing data.

### Conclusions

In spite of the limitations, this study adds to the literature showing that modern information technology can be aligned with conventional face-to-face services. Future studies should investigate the added value of using a support system in psychiatric care. Another option is to evaluate the usability of the support system when training new therapists.

## Abbreviations

BAI: Beck Anxiety Inventory
CBT: cognitive behavioral therapy
GAD-7: Generalized Anxiety Disorder Screener‒7 items
GEE: generalized estimation equations
ICBT: Internet-delivered cognitive behavioral therapy
MADRS-S: Montgomery-Åsberg Depression Rating Scale‒self-rating version
OR: odds ratio
PHQ-9: Patient Health Questionnaire 9 items
QOLI: Quality of Life Inventory
